# Treating Depression and Depression-Like Behavior with Physical Activity: An Immune Perspective

**DOI:** 10.3389/fpsyt.2013.00003

**Published:** 2013-02-04

**Authors:** Harris A. Eyre, Evan Papps, Bernhard T. Baune

**Affiliations:** ^1^Discipline of Psychiatry, School of Medicine, University of AdelaideAdelaide, SA, Australia; ^2^School of Medicine and Dentistry, James Cook UniversityTownsville, QLD, Australia

**Keywords:** physical activity, exercise, depression, psychiatry, immune, neurobiology

## Abstract

The increasing burden of major depressive disorder makes the search for an extended understanding of etiology, and for the development of additional treatments highly significant. Biological factors may be useful biomarkers for treatment with physical activity (PA), and neurobiological effects of PA may herald new therapeutic development in the future. This paper provides a thorough and up-to-date review of studies examining the neuroimmunomodulatory effects of PA on the brain in depression and depression-like behaviors. From a neuroimmune perspective, evidence suggests PA does enhance the beneficial and reduce the detrimental effects of the neuroimmune system. PA appears to increase the following factors: interleukin (IL)-10, IL-6 (acutely), macrophage migration inhibitory factor, central nervous system-specific autoreactive CD4+ T cells, M2 microglia, quiescent astrocytes, CX3CL1, and insulin-like growth factor-1. On the other hand, PA appears to reduce detrimental neuroimmune factors such as: Th1/Th2 balance, pro-inflammatory cytokines, C-reactive protein, M1 microglia, and reactive astrocytes. The effect of other mechanisms is unknown, such as: CD4+CD25+ T regulatory cells (T regs), CD200, chemokines, miRNA, M2-type blood-derived macrophages, and tumor necrosis factor (TNF)-α [via receptor 2 (R2)]. The beneficial effects of PA are likely to occur centrally and peripherally (e.g., in visceral fat reduction). The investigation of the neuroimmune effects of PA on depression and depression-like behavior is a rapidly developing and important field.

The increasing burden of major depressive disorder (MDD; WHO, 2008) makes the search for an extended understanding of etiology, and for the development of additional treatments highly significant. The global “pandemic” of physical inactivity (Lee et al., 2012) – a significant etiological factor for many non-communicable diseases, including depression (Garber et al., 2011; Kohl et al., 2012; Lee et al., 2012) – as well as the growing evidence supporting the clinical utility of physical activity (PA) in many psychiatric disorders, make the biological effects of PA highly relevant (Knochel et al., 2012; Lautenschlager et al., 2012; Rimer et al., 2012). Biological factors may be useful biomarkers for treatment with PA, and neurobiological effects of PA may herald new therapeutic developments in the future.

The neuroimmune system is important in the pathogenesis and pathophysiology of depression-like behaviors (Eyre and Baune, 2012c). Elevations in pro-inflammatory cytokines (PICs), causing neuroinflammation, are well known to be involved in the development of depression-like behaviors – e.g., sickness-like behavior, cognitive dysfunction, and anhedonia – in pre-clinical and clinical populations (Dantzer et al., 2008; McAfoose and Baune, 2009; Miller et al., 2009). The involvement of PICs in the development of depression-like behavior is often referred to as the cytokine model of depression (Dantzer et al., 2008; McAfoose and Baune, 2009; Miller et al., 2009).The neuroinflammatory state is associated with neurotransmitter dysfunction [e.g., reductions in serotonin (5-HT), as well as neurotoxic levels of glutamate (GLU) and tryptophan catabolites], reduced hippocampal (HC) neuroplasticity [e.g., neurogenesis, synaptic plasticity, and long-term potentiation (LTP)], oxidative stress, and glucocorticoid insensitivity (Dantzer et al., 2008; Miller et al., 2009; Eyre and Baune, 2012c; Leonard and Maes, 2012; Moylan et al., 2012).

A variety of novel neuroimmune mechanisms may also be involved in the development of depression-like behaviors (Eyre and Baune, 2012c; Littrell, 2012). Cellular immune factors include various T cells [e.g., CD4+CD25+ T regulatory cells (T regs), CNS-specific autoreactive CD4+ T cells] and macrophages (e.g., M2-type blood-derived macrophages) involved in the model of protective immunosurveillance (Schwartz and Shechter, 2010a,b; Martino et al., 2011; Ron-Harel et al., 2011). These neuroprotective immune cells – found to release neurotrophic factors and anti-inflammatory cytokines (AICs; Schwartz and Shechter, 2010a,b; Martino et al., 2011; Ron-Harel et al., 2011) – may be dysfunctional in the disease state (Schwartz and Shechter, 2010b). Moreover, the function of immunomodulatory proteins such as CX3CL1 (aka fractalkine; Rogers et al., 2011; Corona et al., 2012; Giunti et al., 2012), insulin-like growth factor-1 (IGF-1; Park et al., 2011a), and CD 200 (Lyons et al., 2007; Ojo et al., 2012) may be reduced.

In clinical studies, PA has shown efficacy in the treatment of MDD (Rimer et al., 2012), schizophrenia (SCZ; Knochel et al., 2012), anxiety-based disorders (Asmundson et al., 2013), and in enhancing cognitive function in disorders of cognitive function (i.e., Alzheimer’s disease, AD and mild cognitive impairment, MCI; Foster et al., 2011; Knochel et al., 2012; Lautenschlager et al., 2012). There are many reasons why PA is an attractive therapeutic option in psychiatry. It has a low side-effect profile and can be adapted according to a patient’s medical co-morbidities and functional status (Garber et al., 2011; Knochel et al., 2012; Rimer et al., 2012). PA also enhances self-esteem (Salmon, 2001), has less stigmatization than psychotherapy, may reduce the use of pharmacotherapies in MDD (Deslandes et al., 2010) and has a positive effect on cardio-metabolic risk factors relevant to many psychiatric diseases (e.g., chronic inflammation, visceral fat mass, glucocorticoid sensitivity, glucose control, and insulin sensitivity; Gleeson et al., 2011; Baune et al., 2012c; Hamer et al., 2012; Knochel et al., 2012; Stuart and Baune, 2012).

Physical activity has beneficial effects on depressive symptomatology in a variety of clinical contexts. It is found to have robust effects on the depressive phenotype found in MDD (Rimer et al., 2012), as well as beneficial effects on the depressive symptomatology involved in the negative symptoms of SCZ (Knochel et al., 2012). PA has also been shown to be effective in treating cognitive dysfunction-related depression (Knochel et al., 2012; i.e., in MCI and AD where a significant proportion of patients with AD suffer from co-morbid depression; Lee and Lyketsos, 2003). The clinical utility of PA in MDD is promising given most patients on antidepressants will not achieve remission following initial treatment (Trivedi et al., 2006), and nearly one-third will not achieve remission even following several treatment steps (Rush et al., 2006a,b). Encouragingly, a recent Cochrane meta-analysis of 28 trials (1101 participants) by Rimer et al. (2012) – comparing exercise with no treatment or control intervention – found a moderate clinical effect in MDD. Studies have found that whilst PA has an initial treatment effect equal to that of antidepressants (Rimer et al., 2012), its effects are slower (Blumenthal et al., 1999) with greater relapse prevention (Babyak et al., 2000). PA interventions have been shown to be efficacious as a stand-alone (Rethorst et al., 2009) and as an augmentation treatment for MDD (Trivedi et al., 2011). Adequate levels of PA are also found to have a role in the prevention of MDD (Pasco et al., 2011b).

Physical activity interventions are found to have a multitude of effects on neuroimmune processes (Eyre and Baune, 2012a). Most notably PA interventions are found to reduce PIC levels in the brain of rodents (Eyre and Baune, 2012a) and in the periphery in clinical studies (Beavers et al., 2010a; Rethorst et al., 2012). The anti-inflammatory effects of PA may be related to acute elevations in neuroprotective interleukin-6 (IL-6; Funk et al., 2011), and resultant downstream changes, e.g., increased IL-1ra and reduced neuronal death in the HC (Funk et al., 2011). Reductions in pro-inflammatory visceral fat mass may also play a role in the anti-inflammatory effect of PA (Gleeson et al., 2011).

The neuroimmune effects of PA were recently outlined in our review (Eyre and Baune, 2012a), however, there have been a large number of studies published in 2012 investigating other neuroimmune-related factors (Moon et al., 2012; Rethorst et al., 2012). Novel factors investigated include macrophage migration inhibitor factor (MIF; Moon et al., 2012), CX3CL1 (Vukovic et al., 2012), and IGF-1 (Duman et al., 2009). Taken together, there is a need for a review outlining and summarizing these recent studies in light of pre-existing literature with the intention of better understanding the neuroimmunological effects of PA. From this literature important questions arise: *Are there PA types which are more effective than others? Are there subpopulations of patients with MDD who would benefit more from PA than antidepressants or psychotherapy? Can the neuroimmune effects of PA inform therapeutic development in the future? Are immune biomarkers potentially useful in measuring a treatment effect for PA in depression?*

This paper provides a thorough and up-to-date review of studies examining the neuroimmunomodulatory effects of PA on the brain in depression and depression-like behaviors.

## Methods

This review utilized an electronic search of databases such as PubMed, PsychInfo, OvidSP, and Science Direct. An initial search was conducted using the following keywords: (PA OR exercise) AND (immune OR inflammation OR cytokine OR anti-inflammatory OR immune cell OR glia OR neuroplasticity) AND/OR depression. Abstracts were selected based on the year of publication (between 1995 and December 2012), publication in the English language and of peer-reviewed type. They were excluded if they included anecdotal evidence. A total of 16,000 studies were found using these search terms. A total of 1000 articles remained after assessment of abstracts for relevance to the aims of this review. Of these, 770 studies were excluded after review of the full text if they did not examine the effect of the PA or depression on the immune system. A proportion of papers were found via the reference lists of the 1000 full text articles. Finally, 230 articles were utilized in this review.

## Clinical Efficacy of Physical Activity in Depression

Evidence supporting the clinical efficacy of PA interventions with depression – and depression co-morbid with other diseases [MCI, coronary heart disease (CHD)] – is growing (Blumenthal et al., 2012a,b; Rimer et al., 2012). In the clinical setting, exercise interventions are defined as “planned, structured, and repetitive bodily movements done to improve or maintain one or more components of physical fitness” (Garber et al., 2011). Exercise types can include aerobic, resistance, neuromotor (involving balance, agility, and co-ordination), and flexibility types (Garber et al., 2011). The following section will outline clinical evidence supporting the use of exercise in depression.

A 2012 re-analysis of available clinical trials by the Cochrane Group (Rimer et al., 2012; 2009 version; Mead et al., 2008) revealed 28 trials (1101 participants) comparing exercise with no treatment or control intervention finding a moderate clinical effect in MDD (standardized mean difference, SMD; −0.67 95% CI −0.90 to −0.43). However, when the meta-analysis was conducted with more strict criteria – i.e., studies with adequate allocation concealment, intention-to-treat analysis, and blinded outcome assessment – there were only four trials (326 participants), the SMD indicated a small clinical effect (SMD −0.31 95% CI −0.63 to 0.01). Moreover, data from the seven trials (373 participants) that provided long-term follow-up also found a small effect for exercise interventions (SMD −0.39, 95% CI −0.69 to −0.09). In comparison to cognitive behavioral therapy, six trials (152 participants) found no significant difference with exercise.

Further investigating the individual clinical trials analyzed in this field yields interesting information on the clinical effect of exercise regimens. A 16-week randomized controlled trial (RCT) study by Blumenthal et al. (1999) found aerobic exercise and antidepressant (sertraline) treatment were equally effective in reducing depressive symptom severity [as per both Hamilton Depression Rating Scale (HAM-D) and Beck Depression Inventory (BDI)], however, sertaline had a faster initial response (in the first 3 weeks). Shortly after, a paper by Babyak et al. (2000) was published on the same study participants showing – at 6 months follow-up – patients assigned to the exercise program were less likely to relapse (no longer diagnostic for MDD or HAM-D < 8) than patients assigned to antidepressant treatment. Self-initiated exercise after the study intervention was associated with a reduced probability of depression at the end of the follow-up period (OR = 0.49).

Treatment of depression in older people is often hampered by poor recognition and increased prevalence of medication side-effects, polypharmacy, and poor adherence to treatment; therefore, exercise is increasingly being evaluated as a possible treatment. A recent meta-analysis (Bridle et al., 2012) of seven trials of subjects ≥60 years found exercise was associated with significantly lower depression severity (SMD −0.34; 95% CI −0.52 to −0.17). These findings were irrespective of whether participant eligibility was determined by clinical diagnosis or symptom checklist. An RCT in elderly patients (>60 years) with MDD – non-responders to escitalopram – found a 10-week Tai Chi Chih (TCC) exercise intervention augmented antidepressant treatment (Lavretsky et al., 2011). TCC exercise was chosen given it can be readily implemented among older adults with physical limitations (due to chronic medical illnesses or poor balance) and its added stress reduction and mindful cognitive properties. Multiple studies have shown regular, moderate PA can have a positive influence on depressive symptomatology in subjects with AD (Knochel et al., 2012), however Mahendra and Arkin (2003) found this beneficial effect was only significant after >1 year of PA. Deslandes et al. (2010) reported patients with co-morbid MCI and MDD could significantly reduce their antidepressant dose when they underwent a PA program.

Exercise is shown to have some modest beneficial effects on certain aspects of neurocognitive disturbance in depression. An RCT study with patients who met MDD criteria found exercise (both supervised and home-based) performed better with exercise than sertraline on tests of executive functioning, but not on tests of verbal and working memory (Hoffman et al., 2008). A recent meta-analysis (Smith et al., 2010) examining the effects of aerobic exercise on neurocognitive performance found 29 studies (2049 participants) showing modest improvements in attention and processing speed (*g* = 0.158; 95% CI, 0.055–0.260), executive function (*g* = 0.123; 95% CI, 0.021–0.225), and memory (*g* = 0.128; 95% CI, 0.015–0.241).

Depression is a common co-morbidity with a variety of cardiac conditions. Depression affects as many as 40% of patients with heart failure (HF), with up to 75% of patients reporting elevated depressive symptoms (Blumenthal et al., 2012a). For CHD, MDD affects 15–20% of cardiac patients and an additional 20% report elevated depressive symptoms (Blumenthal et al., 2012b). Blumenthal et al. (2012a) recently published an RCT of 2322 stable HF patients who underwent an aerobic exercise program (supervised for 1–3 months followed by home exercise for 9 months) or education and usual guideline-based HF care. Compared with usual care, aerobic exercise resulted in lower mean BDI-II scores at 3 and 12 months (differences of −0.76 and −0.68, respectively). Another study by Blumenthal et al. (2012b) assessed efficacy of 4 months of aerobic exercise and antidepressant treatments (sertraline) in reducing depressive symptoms and improving cardiovascular biomarkers in depressed patients with CHD. At 4 months, exercise and sertraline were equally as effective at reducing depressive symptoms (HRSD) vs. placebo. Exercise tended to result in greater reductions in heart rate variability vs. sertraline.

When considering the anti-depressive effects of exercise – in addition to biological effects – we must consider psychosocial aspects. Studies have shown exercise regimens have a distraction effect (from negative thoughts and ruminations), provide a sense of mastery via the learning of new skills (Lepore, 1997), and hence enhance self-efficacy (Craft, 2005) and self-esteem (Salmon, 2001). A study by Craft (2005) found that those who experienced an increase in mood following exercise showed higher self-efficacy levels at 3 and 9 weeks post-exercise. Self-esteem is considered to be one of the strongest predictors of overall (Diener, 1984), subjective well-being and low self-esteem is considered to be closely related with mental illness (Fox, 2000). The abovementioned beneficial psychological effects may lead to the stress reducing and stress-resilience enhancing effects of exercise (Salmon, 2001). Additionally, exercise regimens in a group setting may have a beneficial effect via training social skill deficits (Rimer et al., 2012).Therefore, considering the immunomodulatory effects of social support, i.e., social isolation stress is repeatedly shown to enhance inflammation in clinical and pre-clinical models (Hafner et al., 2011), the social interaction effects of PA interventions must be considered as a confounder.

Whilst the vast majority of research using PA in psychiatry is positive and encouraging, it is important to also consider potential pre-cautions during PA interventions. Some studies report no effect for PA in depression (Rimer et al., 2012). This may be explained by inappropriate intensity of PA, or a too short duration of PA as a treatment (Rimer et al., 2012). In order to enhance the potential for antidepressant effects, multiple authors now recommend exercise of moderate-intensity and of at least 8 weeks duration (Mead et al., 2008; Trivedi et al., 2011; Rimer et al., 2012). PA regimens must be tailored according to the individual patient’s functional status and other co-morbidities. Failing to do so can lead to further morbidity and/or mortality. In patients with social phobia-related symptoms, the approach to PA interventions should be tailored appropriately.

## Neuroimmunological Effects of Physical Activity in Depression

When considering the neuroimmunological effects of PA in depression, it is important to first outline the current understanding on neuroimmunological mechanisms of the depression-like disease states. Therefore, the following section will outline these neuroimmunological mechanisms in detail; following, the neuroimmunological effects of PA will be examined.

### Neuroimmunological changes in depression

The neuroimmunological changes found in depression involve humoral and cellular factors from both the innate and adaptive immune systems (Eyre and Baune, 2012c; Littrell, 2012). Humoral factors include PICs, AICs, C-reactive protein (CRP) as well as other immunomodulatory factors like CX3CL1, CD200, and IGF-1 (Eyre and Baune, 2012b). Cellular factors include resident glia (e.g., astrocytes, microglia) and centrally migrating immune cells involved in protective immunosurveillance (e.g., CD4+ T cells and macrophages; Eyre and Baune, 2012b).

#### Neuroinflammation and depression: a well recognized relationship

The neuroinflammatory state is well known to be associated with the depressive phenotype (Dantzer et al., 2008; Dowlati et al., 2010). For example, a recent meta-analysis found a significant correlation between tumor necrosis factor (TNF-α), IL-6, and CRP with depression in humans (Dowlati et al., 2010). Neuroinflammation is characterized by elevations in PICs and reductions in AICs and can arise within the CNS itself, or peripheral inflammatory signals can be transferred into the CNS (Dantzer et al., 2008; see Quan and Banks, 2007; for a review of peripheral-CNS pathways, including: the neural route, circumventricular organs, BBB transport of cytokines, and secretions from BBB cells). The neuroinflammatory state is known to cause neurovegetative or sickness-like symptoms, depression- and anxiety-like behaviors, as well as cognitive dysfunction and symptoms of Chronic Fatigue Syndrome (Dantzer et al., 2008; McAfoose and Baune, 2009; Dowlati et al., 2010; Miller, 2010; Yirmiya and Goshen, 2011; Bansal et al., 2012), and the causation of these phenotypic states by PICs has been modeled in both rodent and human models and extensively reviewed (Dantzer et al., 2008; Miller, 2010).

Neuroinflammation-based models of depression have shown PICs to impact on other major neurobiological systems involved in depression. Neuroinflammation affects the neurotransmitter systems by activation if the tryphophan degrading enzyme, indoleamine 2,3 dioxygenase (IDO), altering metabolism of tryptophan into neurotoxic metabolites (3-hydroxykyurenin, 3-HK and quinolinic acid, QA) and depleting its availability for serotonin (5-HT) synthesis (Miller, 2010; Dantzer et al., 2011; Moylan et al., 2012). Inflammation also stimulates the reuptake of monoamines from the synapse by increasing the activity and the density of 5-HT, noradrenaline, and dopamine transporters (Moron et al., 2003; Nakajima et al., 2004; Zhu et al., 2006). Evidence suggests these immune mechanisms adversely affected glutamatergic neurotransmission causing GLU to rise to neurotoxic levels (McNally et al., 2008; Hashimoto, 2009; Popoli et al., 2012). In the neuroinflammatory state PICs may disrupt the capacity of the glucocorticoid receptor to translocate to the nucleus where it normally acts to suppress the activity of pro-inflammatory transcription factors such as nuclear factor-kappa B (NF-κB) – this is termed glucocorticoid resistance (Dantzer et al., 2008; Miller, 2010; Muller et al., 2011). High levels of PICs impair processes of neuroplasticity in the HC, such as neurogenesis, LTP, neurotrophin production (e.g., brain-derived neurotrophic factor, BDNF), and synaptic plasticity (Miller, 2010; Eyre and Baune, 2012c). In the context of reduced neuroplasticity, elevations in neurotoxic oxidative stress products and markers of apoptosis are found in the HC (Moylan et al., 2012). An in-depth assessment on the effects of inflammation on these systems is outside the scope of this review and have been outlined recently (see Dantzer et al., 2008, 2011; McAfoose and Baune, 2009; Muller et al., 2011; Moylan et al., 2012).

#### Rationale for examining immune mechanisms in addition to inflammation

Whilst the cytokine and neuroinflammatory models of depression have been helpful in understanding the neurobiology behind the depressive phenotype, there are a number of clinical and biological reasons for investigating neuroimmune mechanisms in addition to inflammation. These factors include:

- A recent meta-analysis by Hannestad et al. (2011) found results arguing against the notion that resolution of a depressive episode is associated with normalization of levels of circulating PICs. This analysis of 22 studies (603 subjects) found – when all antidepressants were grouped – these medications reduced levels if IL-1β with a marginal effect on IL-6 (using less stringent fixed-effects models); there was no effect on TNF-α. However, a sub-group analysis of selective serotonin regulate inhibitors (SSRI) medication found a reduction in IL-6 and TNF-α. Other antidepressants did not reduce PIC levels.
- Recent evidence has emerged to suggest no effect or even an antagonistic effect for anti-inflammatory medications in depression. A large-scale prospective cohort study of treatment-resistant depression, the “sequenced treatment alternatives to relieve depression” (STAR*D), found an antagonistic effect for anti-inflammatory compounds on ADs (Warner-Schmidt et al., 2011). Patients reporting concomitant non-steroidal anti-inflammatory drug (NSAID) or other analgesic treatment showed a reduced therapeutic response to citalopram, hence, the authors suggest concomitant use of NSAIDs may be an important reason for high SSRI treatment resistance rates (Warner-Schmidt et al., 2011). A recent re-analysis reached a similar conclusion, with more modest effects persisting after adjustment for potential confounding variables (Gallagher et al., 2012). Another recently published study shows no difference between infliximab, a TNF-α antagonist, and placebo in a recent 12-week double-blind, placebo-controlled RCT for treatment-resistant depression (Raison et al., 2012). There was a significant effect for infliximab in individuals who had a high baseline hs-CRP (>5 mg/L) and a significant effect for placebo-treated patients at a baseline hs-CRP of <5-mg/L. Schwartz and Shechter hypothesize anti-inflammatory drug compounds may block the production of brain-derived cytokines and chemokines which promote the migration of neuroprotective immune cells involved in protective immunosurveillance toward the CNS (Schwartz and Shechter, 2010b; Warner-Schmidt et al., 2011). Importantly, however, the use of NSAIDs may be most useful when used in the correct stage of neuroinflammatory diseases, i.e., administered early in the neuroinflammatory disease course when transmigratory immune cells have not come into effect (Schwartz and Shechter, 2010b).
- Evidence is emerging to suggest a neuroprotective and physiological role for “PICs.” TNF-α and IL-6 have been shown to play an integral roles in processes of memory and learning in both human and rodent studies, as well as having a physiological role in HC neuroplasticity (Carlson et al., 1999; Eyre and Baune, 2012c). The *TNF-α* gene (*rs1800629*) is correlated with enhanced cognitive processing speed in a healthy human population (Baune et al., 2008a). The *IL-6* gene (*rs1800795*) has been correlated with increased to HC volume in a healthy human population (Baune et al., 2012a). There are other studies outlining a neuroprotective effect of PICs in the brain (see below).
- From a clinical disease course perspective, there are other mechanisms in depression – in addition to inflammation – which may have a role in explaining the absence of correlation between the increase in neuroinflammation in aging and rates of depression. Since aging itself is related to higher levels of systemic inflammation and neuroinflammation (Hein and O’Banion, 2012), this should lead to higher rates of depression in old age, however, rates are highest in those aged 25–45 years, not in old age (Kessler et al., 2005). Other neuroimmune factors which may explain this scenario will be outlined below.

### Dysfunction of neuroprotective immune factors in depression

When considering neuroimmunological factors in depression, historically the focus has mainly been on high levels of PICs and their detrimental effects on the brain. However, research is beginning to suggest a significant role for neuroprotective neuroimmune factors in depression and other neurobiological disorders (e.g., multiple sclerosis and AD; Martino et al., 2011; Kokaia et al., 2012). When considering these neuroprotective factors in depression, their loss of function may exacerbate the depression-like behaviors (Schwartz and Shechter, 2010b). The following section will outline evidence suggesting a possible beneficial role for a variety of neuroimmune factors.

#### Neuroprotective and physiological effects of cytokines

There are a number of cytokines are found to have neuroprotective and physiological effects.

Interleukin-6 has been found to have neuroprotective effects via gp130 signaling and related pathways [i.e., Janus Kinase (JAK)/Signal Transducer and Activator of Transcription (STAT), Mitogen-activated Protein Kinase (MAPK)/cAMP Response Element-binding (CREB), Ras-MAPK, Phosphatidylinositol 3-kinases (PI3K); Baune et al., 2012a]. These mechanisms affect the production of neurotrophic factors, cellular survival, and apoptosis (Baune et al., 2012a). A recent imaging genetics study investigated the association between the *IL-6* gene and brain morphology in a large cohort of healthy adult participants in a whole-brain analysis approach (Baune et al., 2012a). Carriers of the G-allele of the *IL-6* genetic variant *rs1800795 (-174 C/G)* showed a significant association with larger HC volumes on the right side in healthy subjects. This genotype effect was remarkably specific to the HC, with no other structure surviving statistical threshold for the entire brain. The findings are suggestive of a neuroprotective role of the *IL-6* gene [*rs1800795 (-174 C/G)*] on HC morphology. Supporting a role of IL-6 in neuroproliferation is an *in vivo* study showing that IL-6 knock-out mice have reduced proliferating NSCs specifically in the HC, hence underlining the importance of IL-6 in cell proliferation and cell survival (Bowen et al., 2011). However, other similar studies have shown no effect or a negative effect for IL-6 in neurogenesis processes (Eyre and Baune, 2012c). The difference between the pro- and anti-neurogenic effects of IL-6 may reflect differences in amounts and conditions used experimentally (Eyre and Baune, 2012c).

Tumor necrosis factor-α is thought to exert its protective and restorative effects primarily via TNFR2 (p75; primarily neuroprotective and neuroregenerative pathway) and related signaling pathways [i.e., IκB kinase (IKK)/Nuclear Factor κB (NF-kB), Transforming Growth factor β-activated Kinase 1 (TAK-1), PI3K-PKB-Akt, c-Jun N-terminal kinases (JNK), and IL-6), as opposed to the TNFR1 (p55; primarily neurodegenerative; Eyre and Baune, 2012a; Santello and Volterra, 2012). Importantly, whether the outcome of TNF-α signaling is protective or damaging may depend upon duration of NF-κB activation (Santello and Volterra, 2012). TNF-α has been found to exert beneficial effects in depression-related processes, e.g., cognitive function and HC neurogenesis (Eyre and Baune, 2012a; Santello and Volterra, 2012). During relatively health aging processes, it has been shown that the *TNF*-α gene (*rs1800629*) has protective effects on cognitive processing speed (Baune et al., 2008a) and has been associated with cognitive processes (e.g., response inhibition, error processing, attentional processes, and mental rotation) in young health individuals (Eyre and Baune, 2012a). In behavioral studies, TNF-α deficient mice exhibit impaired HC-dependent memory function in the Morris Water Maze suggesting that during early stages of brain development basal levels of TNF is required for memory and learning (Baune et al., 2008b).

Interleukin-4 has been found to have a beneficial role in depression-like behaviors and a neuroprotective effect. The release of IL-4 from CNS-specific autoreactive CD4+ T cells involved in protective immunosurveillance – in response to increased neurotoxicity (Ron-Harel et al., 2011) – binds to IL-4 receptor on the cytotoxic microglia (Kipnis et al., 2008) causing downregulation of PIC production, induction of BDNF and IGF-1, and an elevation in neurogenesis (Butovsky et al., 2005, 2006b; Lyons et al., 2009; Martino et al., 2011). Microglia under quiescent conditions, after exposure to IL-4 or low levels of IFN-γ (Butovsky et al., 2006b), have been shown to support neurogenesis and NSC differentiation and migration *in vitro* (Aarum et al., 2003; Butovsky et al., 2006b; Walton et al., 2006). IL-4 is also shown to promote the creation of neuroprotective M2-type microglial phenotype (Godbout et al., 2012). A recent study found central IL-4 administration increased microglial-specific M2a-type genes including *Arginase*, *IL-1R*α, and *BDNF* (Godbout et al., 2012). Microglia activated by IL-4 remain committed to their protective phenotype (M2-type) even when exposed to a threatening environment in the form of LPS, and, exposure of microglia, pre-activated to a cytotoxic phenotype, to IL-4 induces a phenotype switch toward neuroprotection (Butovsky et al., 2005; Schwartz et al., 2006). A study rodent by Derecki et al. (2010) shows T cell-derived IL-4 to have beneficial effects on the regulation of cognitive function in rodents via meningeal myeloid cell phenotypes producing BDNF. IL-4 knock-out mice show greater sickness behavior (measured by exploratory behavior) than wildtype mice exposed to LPS (Lyons et al., 2009). Interestingly, Kim et al. (2011) proposes T-bet deficient mice may have a neuroprotective effect by creating a predominance of Th2-derived IL-4, which may in turn stimulate meningeal myeloid cell BDNF production. T-bet is a Th1-specific T-box transcription factor which regulates CD4+ Th1 development by inducing endogenous Th1 cytokines, while simultaneously repressing Th2 development (Wong et al., 2008).

A role for IL-10 in neuroprotection and the prevention of depression-like behavior has been suggested. Central administration of IL-10 prevents the emergence of behavioral signs of depression in an LPS model of sickness behavior (Bluthe et al., 1999). IL-10 over-expression mice display less anxiety-like behaviors, while IL-10 knock-out rodents display greater anxiety and depression-like behavior (forced-swim test) with these effects more pronounced in females (Mesquita et al., 2008). In human studies, IL-10 is found to be reduced in the depressed state (Himmerich et al., 2010). Further papers examining the neuroprotective effects of IL-10 can be found in Raison and Miller (2011).

#### Immunomodulatory factors

Insulin-like growth factor-1 is a major neurotrophic factor involved in neuroplastic functions such as neurogenesis and is critical in normal memory and LTP functions (Trejo et al., 2007). Recent evidence suggests IGF-1 also has added immunomodulatory effects (Park et al., 2011a,b). In an LPS model of depression, central administration of IGF-1 is shown to prevent LPS-induced sickness- and depression-like behavior (Park et al., 2011a,b) in association with an induction of BDNF and a reduction of TNF-α, IL-1β, and iNOS in the pre-frontal cortex (PFC; Park et al., 2011b). Given the levels of IGF-1 have been found to be low in rodent models of depression (Mitschelen et al., 2011), the absence of this anti-inflammatory factor may exacerbate the neuroinflammatory and anti-neuroplastic state in depression.

CX3CL1 is a chemokine expressed by healthy neurons which has its receptor, CX3CR1, in membrane bound form or as soluble ligand (Rogers et al., 2011). It has an important role in inhibiting the activation of microglia (Rogers et al., 2011). A recent study with CX3CR1 knock-out mice and the LPS model of sickness behavior found a deficiency in the action of CX3CL1 resulted in protracted microglial activation, as measured by IL-1β and CD14 (Corona et al., 2010). These mice have extended LPS-induced depression-like behavior in association with the activated microglial phenotype described (Corona et al., 2010). In another study with CX3CR1 knock-out mice, a lack of the CX3CR1 receptor resulted in contextual fear conditioning (associative memory) and Morris Water Maze deficits (spatial memory), as well as impairment in LTP (Rogers et al., 2011). Disruption of the CX3CL1/CX3CR1-pathway in young rodents decreases both survival and proliferation of HC neural progenitor cells (Bachstetter et al., 2011).

CD200 is a membrane glycoprotein which has been identified as an immune-suppressive molecule (Cox et al., 2012). It is expressed in neurons and oligodendrocytes, but not on microglia (Cox et al., 2012). The receptor for CD200, CD200R, is also a membrane glycoprotein and is primarily restricted to cells of the myeloid lineage, hence being found on microglia, but not neurons or astrocytes (Cox et al., 2012). The interaction between CD200 and its receptor play a significant role in maintaining microglia in a quiescent state, therefore, a decrease in CD200 expression is associated with evidence of microglia activation (Cox et al., 2012). A rodent study by Frank et al. (2007) shows an inescapable shock model of stress over 24 h resulted in a downregulation of HC CD200 in association with enhanced LPS-induced cytokine production in HC microglia. This suggests stress can activate microglia via downregulation of CD200, enhancing the PIC production of microglia (Frank et al., 2007). A study by Cox et al. (2012) found a CD200 fusion protein (CD200Fc), activator of CD200R, attenuated age-related microglial immunoreactivity in the HC (indicated by MHCII, CD40, and iNOS). CD200Fc also attenuated LPS-induced microglial activation (indicated by elevated MHCII, CD40, CD11b, and CD68) and LTP deficits (Cox et al., 2012). Using CD200 knock-out mice and LPS-induced sickness behavior, Costello et al. (2011) found the neuroinflammatory changes resulting from CD200 deficiency have a negative impact on LTP in the CA1 region of the dentate gyrus. Interestingly, a study by Lyons et al. (2009) has shown IL-4 as a key inducer of CD200 expression.

#### Dysfunction of protective immunosurveillance

Emerging data suggests a role for CNS-specific autoreactive CD4+ T cells, blood-derived macrophages (in the form of M2 alternatively activated macrophages) in physiological, protective immunosurveillance functions of the brain (Derecki et al., 2010, 2011; Martino et al., 2011; Ron-Harel et al., 2011). Evidence suggests these cell types may have established a physiological connection between the immune system and the brain, and have assisted in explaining processes of HC-dependent neurogenesis and cognitive dysfunction (Kipnis et al., 2004b; Butovsky et al., 2006b, 2007; Ziv et al., 2006; Brynskikh et al., 2008; Derecki et al., 2010, 2011), anxiety- and depression-like behavior (Cohen et al., 2006; Lewitus et al., 2008; Cardon et al., 2010) due to an insufficient immune response (Derecki et al., 2010, 2011; Schwartz and Shechter, 2010a,b; Ron-Harel et al., 2011). The role of these cells in neuroprotection and higher neurocognitive functions has been reviewed in detail elsewhere (Martino et al., 2011; Yirmiya and Goshen, 2011); however, a brief summary will be given, below.

Immune cells involved in protective immunosurveillance can populate meningeal areas of the choroid plexus and the cerebrospinal fluid, hence gaining access to the healthy brain without entering the parenchyma (Ransohoff et al., 2003; Derecki et al., 2010, 2011; Schwartz and Shechter, 2010a). CNS-specific autoreactive CD4+ T cells are suggested to react to three signals, (1) T-cell receptor (TCR; Ron-Harel et al., 2011), (2) co-stimulatory signals (CD28/CD80,86; Jenkins and Johnson, 1993), and (3) PICs and reactive oxygen species (ROS; Curtsinger et al., 1999; Tse et al., 2007; Ron-Harel et al., 2011). The T cells in question, activated in response to increased neurotoxicity (Ron-Harel et al., 2011), are thought to secrete increased levels of IL-4 (Ron-Harel et al., 2011), where IL-4 penetrates the brain parenchyma and binds to IL-4R on the cytotoxic microglia (Kipnis et al., 2008). Exposure of cytotoxic microglia to IL-4 causes downregulation of PIC secretion, induction of BDNF and IGF-1 secretion, and an elevation in neurogenesis (Butovsky et al., 2005, 2006b; Martino et al., 2011). All of these signals support the restoration of brain homeostasis (Ron-Harel et al., 2011). Furthermore, the T cells boost infiltration of neuroprotective blood-borne monocytes upon need (Shechter et al., 2009). A recent commentary by Ron-Harel et al. (2011) suggests any destabilization in brain homeostasis that cannot be locally contained by microglia and/or astrocytes will increase T cell recruitment as well as subsequent IL-4 release and recruitment of blood-derived macrophages.

According to the “protective immunosurveillance” model, increased susceptibility to mental illness may result from a deficiency in circulating T cells and the IL-4 they can produce, as the IL-4 mediates processes which are able to counteract neuroinflammation and restore brain homeostasis (Ron-Harel et al., 2011). Indeed, the brains of immune-deficient mice show accumulation of toxicity (i.e., increased glyoxalase-1, a compensatory mechanism against free radical and carbonyl levels; Ron-Harel et al., 2011).

According to the protective immunosurveillance model, activation of CNS-specific autoreactive CD4+ T cells (mentioned above) support the infiltration of neuroprotective, alternatively activated M2 macrophages to the sub-arachnoid meningeal spaces and choroid plexus, via IL-4 and IFN-γ secretion (Derecki et al., 2010, 2011; Ron-Harel et al., 2011). These infiltrating macrophages, together with the microglia they regulate, remove dead cells and cellular debris, buffer toxic compounds (such as GLU and ROS), and produce growth factors (i.e., BDNF and IGF-1), while downregulating inflammation-associated compounds such as IL-1β, TNF-α, iNOS, and COX-2 (Hauben et al., 2000; Butovsky et al., 2005, 2006a,b, 2007; Shaked et al., 2005; Beers et al., 2008; Chiu et al., 2008; Rolls et al., 2008; Shimizu et al., 2008; Koronyo-Hamaoui et al., 2009; Shechter et al., 2009; Derecki et al., 2010, 2011; Prinz et al., 2011). These neurobiological functions are thought to contribute to blood-derived macrophages support of learning and memory (as determined via the Morris Water Maze and Barnes Maze; Derecki et al., 2010, 2011). Importantly, intravenous injection of M2 cells into immune-deficient mice can circumvent the need for CNS-specific autoreactive CD4+ T cells (Derecki et al., 2011). For a review of the role of blood-derived macrophages see recent papers (Derecki et al., 2010; Martino et al., 2011; Yirmiya and Goshen, 2011).

The type of macrophage – classical (M1), alternatively activated (M2), and deactivated types – determines the role in sickness behavior (for thorough review see Moon et al., 2011). Classical macrophages produce PICs and, hence, induce sickness behaviors (Dantzer et al., 2008; Moon et al., 2011). M2 macrophages which reduce PIC production, as outlined above, are associated with a reduction in sickness behavior (Derecki et al., 2010, 2011; Sherry et al., 2010). Deactivated macrophages which inhibit PIC production via IL-10 secretion are also thought to have beneficial effects of sickness behaviors, however, this finding has not been replicated (Moon et al., 2011).

It is important to mention a recent critique of the protective immunosurveillance concept recently produced by Rook et al. (2011). One important issue raised is that the phenotype of the neuroprotective, CNS-specific autoreactive CD4+ T cells is poorly understood (Rook et al., 2011). The authors suggest immune cells involved with the function of protective autoimmunity is likely from a regulatory cell – not always CD25+ – given the involvement of IL-4 and IL-10. Suggestions for potential cell types include Th3, Tr1, Th2, IL-10^+^TH1, CD8+ reg cells, regulatory Foxp3+ NKT, IL-10^+^CD56^bright^NK, or various other IL-10-secreting cell types (Fujio et al., 2010; Rook et al., 2011). Another important consideration raised is the effect of T cell produced IL-4 on T reg differentiation. T cell differentiation into the T reg cell type can be enhanced or opposed by IL-4 depending on the context (Chapoval et al., 2010; Rook et al., 2011). Further, one study shows IL-4 increased certain chemokines (CCL1, CCL17, and CCL22) in an experimental autoimmune encephalitis (EAE) model capable of recruiting T regs (Butti et al., 2008). The above mentioned issues are relevant to the neuroimmune model of depression considering the dynamic relationship between T regs and effector T cells.

#### Glial cells

The role of the immunocompetent glia, astrocytes, and microglia, in depression is complex and poorly understood (Beumer et al., 2012); importantly, however, there is a developing literature supporting a neuroprotective effect of these cells under certain conditions (Schwarz and Bilbo, 2011, 2012; Ekdahl, 2012). The follow section will summarize most recent evidence available in this field.

#### Microglia

The function of microglia is dynamic even in the resting state whereby they continually survey their microenvironments by extending and contracting processes into nearly synapses (Bilbo et al., 2012). Microglia are the resident macrophages of the CNS and are recognized as the primary component of the neuroimmune system (Ekdahl, 2012). Once activated – by chronic stress conditions, or immune challenge with LPS or PICs – microglia are capable of producing PICs and neurotoxic mediators such as nitric oxide, PGE2, and superoxide anions (Liu et al., 2011; Bilbo et al., 2012; Ekdahl, 2012). A recent study by Walker and colleagues has shown a role for microglia in mediating the effects of stress on PFC neuronal function and PFC-regulated behavior (Hinwood et al., 2012). This study found restraint stress conditions caused a decline in working memory performance associated with increased microglial activity (measured by a 25% increase in Iba-1 labeling, ΔFosB, and a hyper-ramified state) in the medial PFC and no association was found with increased antigen presentation (MHCII) or apoptosis (caspase-3; Walker et al., 2011).

Given the pre-existing association with the inflammatory hypothesis of depression much research centers on reducing the PIC production of microglia (Liu et al., 2011). Recent evidence suggests a neuroprotective function of microglia under certain circumstances (Yirmiya and Goshen, 2011; Ekdahl, 2012). For example, microglia under quiescent conditions, after exposure to IL-4 or low levels of IFN-γ (Butovsky et al., 2006b), have been shown to support neurogenesis and NSC differentiation and migration *in vitro* (Aarum et al., 2003; Butovsky et al., 2006b; Walton et al., 2006). Microglia activated by IL-4 remain committed to their protective phenotype even when exposed to a threatening environment in the form of LPS, and, exposure of microglia pre-activated to a cytotoxic phenotype to IL-4 induces a phenotype switch toward neuroprotection (Butovsky et al., 2005; Schwartz et al., 2006). Exposure of rats to environmental enrichment (EE) increases neurogenesis alongside increased HC microglia proliferation (microglia assumed a neuroprotective phenotype expressing MHC II and IGF-1; Ziv et al., 2006). As mentioned previously, the pro-neurogenic effects of microglia may be related to their interactions with CNS-specific autoreactive CD4+ T cells, this was further confirmed by a study showing transgenic mice with an excess of these T cells – and associated increases in neurogenesis – showed attenuated neurogenesis by chronic treatment with the microglial inhibitor, minocycline (Ziv et al., 2006). Furthermore, a rodent model of amyotrophic lateral sclerosis (ALS) illustrates the interaction between T cells and microglia whereby Th1 cytokines promote M1 microglia and Th2 or Treg cytokines promote M2 microglia (Chiu et al., 2008). Microglia were also shown to support neurogenesis in adrenalectomized rodents via TGF-β (Battista et al., 2006; Mathieu et al., 2010). Opposing the above neuroprotective findings is a rodent study demonstrating that PA-induced neurogenesis was not associated with microglial proliferation or activation, and no indication of T-cell-microglial interactions (i.e., no MHC II expression or T cells in the HC; Olah et al., 2009).

In summary, microglial function is closely intertwined with the immune system and neurogenesis (Ekdahl, 2012), with the cross-talk between these systems requiring further investigation. For instance, a recent review by Ekdahl (2012) suggests microglial activation patterns may by region-specific. Moreover, there appears to be a primarily beneficial interaction between microglia and new neurons in the intact brain, however, the cross-talk is complex and probably double-edged in pathological conditions, especially following long-term microglial activation (Ekdahl, 2012).

#### Astrocytes

Astrocytes are physically and functionally appositioned with most synapses, known as the “tripartite synapse” (Araque et al., 1999). They possess immune-like properties whereby they have an ability to respond to inflammatory cytokines (particularly IL-1β), to secrete PICs (i.e., TNF-α and IL-6) and to phagocytose cellular processes and debris (Yirmiya and Goshen, 2011). These cells play an important role in neural and synaptic functioning. For example, a rodent study by Bracchi-Ricard et al. (2008) shows female mice where the transcription factor NF-κB was inhibited specifically in astrocytes displayed deficits in learning, memory, and LTP. These cells were also found to mediate homeostatic synaptic scaling following prolonged inhibition of neuronal activity via TNF-α secretion, a known synaptic strength enhancer (Stellwagen and Malenka, 2006; Kaneko et al., 2008). The role of astrocytic IL-1 signaling in memory functioning and LTP was recently demonstrated by Ben Menachem-Zidon et al. (2011). In this study neural precursor cells (NPCs) derived from either WT or IL-1rKO neonatal mice were labeled with BrdU and transplanted into the HC of either IL-1rKO or WT adult host mice. Transplanted NPCs showed long-term survival and differentiated into astrocytes (expressing GFAP and S100β), but did not differentiate into neurons. Several weeks post-transplantation, IL-1rKO mice transplanted with IL-1rKO cells, or sham operated, displayed severe memory disturbances and a marked impairment in LTP. However, IL-1rKO mice transplanted with WT NPCs (expressing IL-1R) displayed complete rescue of the impaired memory functioning, as well as partial restoration of LTP. IL-4 is also found to be important in astrocyte functioning with the secretion of BDNF by *in vitro* astrocytes being markedly enhanced by this cytokine (Martino et al., 2011). Furthermore – and in fitting with the abovementioned model of protective immunosurveillance by Schwartz et al. – astrocytes also acquire a neuroprotective phenotype following their co-culture with T cells (Garg et al., 2008).

There is a paucity of evidence correlating the role of the abovementioned glial cells in models and tests of depression-like behavior. This is an important area for future research as these cells appear to be involved in depression-related pathophysiological processes.

#### Additional cellular immune factors

The role of T regs in depression is uncertain, and may be both positive and negative in depression pathophysiology depending on the surrounding environment (Cohen et al., 2006; Himmerich et al., 2010). In relation to the positive effects of T regs, some authors propose these cells may function to inhibit inappropriate or excessive immune responses, i.e., PIC production (Dantzer et al., 2008; Miller, 2010). Some human studies have found reduced IL-10 and TGF-β have been found in depressed patients, and are thought to be consistent with reduced T reg expression and/or function (Myint et al., 2005; Sutcigil et al., 2007; Dhabhar et al., 2009; Musil et al., 2011). One study found decreased T regs, alongside intracellular Foxp3, in association with IL-10 and TGF-β in depressed patients vs. controls (Li et al., 2010). A second study found 6 weeks of AD treatment led to increased T reg (CD4+CD25^hi^) percentage in association with decreased IL-1β (Himmerich et al., 2010). A recent rodent study shows T reg cell depleted mice undergoing chronic immobilization stress displayed markedly increased anxiety in the Elevated Plus Maze and increased depression-like behavior in the Forced-Swim Test (Kim et al., 2012). These finds were found in correlation with elevated serum cytokines (i.e., IL-6, TNF-α, IL-2, IFN-γ, and IL-4) and reduced levels of HC 5-HT. In addition, a rodent model of cholestatic liver disease due to bile duct ligation found T regs suppress sickness-like behavior alongside inhibiting monocyte and hepatic IL-6 production, and subsequent signaling via circulating IL-6 acting (via p-STAT3 at the level of the cerebral endothelium; Nguyen et al., 2012). However, T regs have also been found to inhibit the beneficial effects of CNS-specific autoreactive CD4+ T cells on mitigating stress-induced anxiety-like behaviors in rodents (Cohen et al., 2006). This suggests T regs may inhibit the neuroprotective functions of these autoreactive T cells, a counterproductive effect. Interestingly, other studies with an optic nerve injury model have shown both Treg-free CD4+ T cells and T regs, respectively, can exhibit neuroprotective functions via preventing neuronal cell loss (Kipnis et al., 2004a). T regs exhibit significant plasticity and can lose regulatory activity, expressing effector cell function under certain circumstances (Zhou et al., 2009). Therefore, the balance of these two cells types may play a role in neuroprotective functions. Interestingly, T regs constitutively express CD25, a high affinity IL-2 receptor. The expression of CD25 is thought to be one of the ways by which T regs suppress proliferation of T effector cells, that is, by acting as a sink for IL-2 which is needed for T effector cell proliferation (Walsh and Kipnis, 2011). Interestingly, IL-2 is known to increase the suppressive abilities of T regs (Kohm et al., 2006), hence, the reduction of IL-2 which is seen in some studies of depression may reduce the anti-inflammatory effects of T regs (Anisman et al., 1999; Blume et al., 2011). A recent review paper summarizes literature suggesting T reg phenotypes are flexible depending on background chemokine and cytokine levels (Rook et al., 2011). Flexibility of phenotype means these cells can change from anti- to pro-inflammatory functions (Rook et al., 2011); indeed, authors remark that T-cell phenotype may change from the start to the end of studies (Rook et al., 2011). Furthermore, gut microbiota may affect the immunosuppressive function of T regs as well as their effects on higher neurocognitive behaviors of the brain (Rook et al., 2011). Clearly, the effect of T regs in depression requires further research.

#### Other T-cell subtypes in depression – Th1, Th2 cells, and T-bet

The balance of Th1 vs. Th2 cytokines in depression is currently debated by prominent authors in the field (Capuron and Miller, 2011; Rook et al., 2011). The majority of evidence suggests a net Th1 production as a key feature of immune dysfunction in depression, however, some studies suggest increased Th2 production (Myint et al., 2005; Capuron and Miller, 2011; Rook et al., 2011; Leonard and Maes, 2012). Th1 cells can produce IFN-γ, IL-2, and TNF-α; Th2 cells can produce IL-4, IL-6, and IL-10. Recent evidence suggests T-bet is associated with depression-like behaviors (Wong et al., 2008; Kim et al., 2011). T-bet deficient mice, Th1/IFN-γ depleted, are shown to be resistant to stress-induced depression-like behavior and stress-induced neuroinflammation (i.e., IL-6 and TNF-α; Kim et al., 2011). A clinical study by Wong et al. (2008) in a sample of Mexican Americans with major depression, shows evidence that single nucleotide polymorphisms (SNPs) in the *T-bet* (*Tbx21*) gene, which is critical for helper T (Th) 1-cell function, are associated with susceptibility to major depression. Moreover, the same study showed T-cell involvement in AD treatment response of genes associated with T-cell development (T-cell antigen receptor-ε subunit of T3, CD3E; Wong et al., 2008).

### Balancing beneficial and detrimental effects of the neuroimmune system in depression

**Figure 1:**
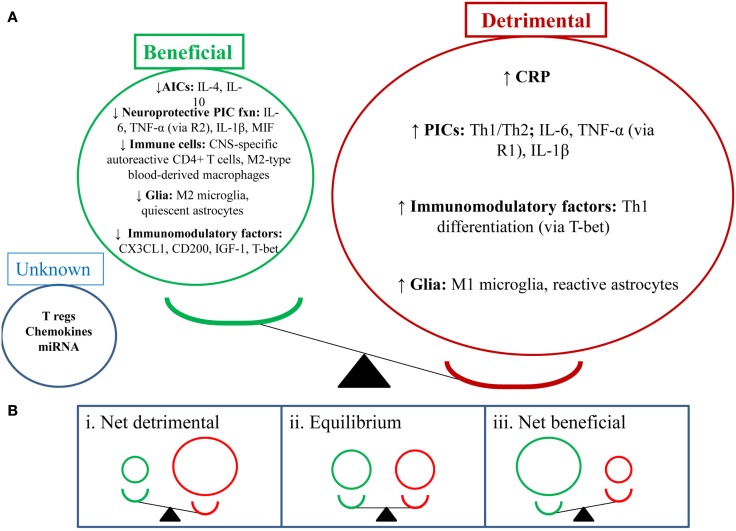
**Depression-like behavior: balancing the beneficial and detrimental effects of the neuroimmune system**. **(A)** This section shows the balance of the detrimental (red) and beneficial (green) effects of neuroimmune factors in the depressed state (i.e., detrimental factors out way beneficial factors). NB: depression-like behavior includes sickness-like behavior, anhedonia, anxiety-, and cognition-like behaviors. **(B)** This section shows a number of potential outcomes for the balance between the abovementioned neuroimmune factors. (i) Shows a net detrimental effect which would lead to depression-like behaviors; clinically this could mean a depressive episode and could also increase relapse rates. (ii) Shows an equilibrium position which may suggest a stable/steady state in behavior; clinically this could mean a euthymic state. (iii) Shows a net beneficial effect which may attenuate depression-like behavior; clinically this could mean reduction or resolution of depressive symptoms and reduced relapse rates.

In the sections above we have outlined both the beneficial and detrimental effects of the neuroimmune system in depression. From this information, we suggest that depression-related pathophysiology and depression-like behaviors may be dictated by the balance between the beneficial and detrimental effects of neuroimmune factors. See Figure 1 for a graphical representation of this balance. It is possible that when the balance is skewed toward the detrimental effects of the neuroimmune system, this leads to the development of depression-like behaviors, may prolong depressive episodes and lead to more severe symptomatology and behaviors. Alternatively, if the balance becomes skewed toward the beneficial effects of the neuroimmune system, this would reduce symptomatology and behavior and may drive the end of depressive episodes and prolong relapse remission.

### Neurobiological effects of physical activity in depression

The neurobiological effects of PA in depression include effects on neurotransmitter, neuroendocrine systems, effects on neuroplasticity, and effects on neuroimmunological factors. The following section will outline the effects of PA on these systems, below, with a focus on neuroimmunological factors.

#### Neurobiological effects

Physical activity has been shown to upregulate monoamine neurotransmitters in the brain (e.g., 5-HT, dopamine, and noradrenaline) as well as endorphins (Knochel et al., 2012; Lautenschlager et al., 2012; Sarris et al., 2012). Reductions in glucocorticoid stress hormones are also found alter PA interventions whereby PA appears to re-regulate the HPA axis (Eyre and Baune, 2012c). Oxidative stress is reduced in the hippocampus in pre-clinical populations (Marosi et al., 2012).

Hippocampal neuroplasticity (e.g., neurogenesis, HC volume, and neurotrophin production) is increased with PA interventions in both clinical and pre-clinical populations (Erickson et al., 2012; Knochel et al., 2012; Lautenschlager et al., 2012). Pereira et al. (2007) reported that aerobic exercise resulted in increased HC blood volume which correlated with improved aerobic capacity and neurogenesis in the dentate gyrus. A recent RCT by Erickson et al. (2011) found that an aerobic exercise program in older adults, for 3 days a week over 1 year, increased HC volume by 2%. This was associated with increased serum BDNF and improvements in spatial memory. Further work is required to investigate the effects of PA on neuroplasticity in the PFC and amygdala.

#### Neuroimmunological effects in clinical populations

A recent study by Rethorst et al. (2012) aimed to determine the extent to which inflammatory markers can be used to predict treatment response to exercise treatment, and if this effect was dependent upon the dose of exercise. This prospective study used participants who were incomplete responders to an SSRI and randomized them to two doses of aerobic exercise for 12 weeks [4 or 16 kilocalories per kilogram of body weight per week (KKW))] 16 KKW was designed to meet or exceed current PA guidelines for public health from professional associations. The study found participants with a high baseline TNF-α (>5.493 pg/ml) had a greater reduction in depressive symptoms (measured by IDS-C) than those with a low TNF-α level. Interestingly, this finding may suggest TNF-α as a moderator between SSRI and exercise treatment, and TNF-α levels could be used to recommend exercise rather than medication as part of a personalized treatment algorithm (Rethorst et al., 2012). This is given Eller et al. (2008) found high baseline TNF-α associated with non-response to an SSRI, and the Hannestad et al. (2011) meta-analysis also supports this association. There was a significant correlation between change in IL-1β and depression symptoms for the 16 KKW group, but not the 4 KKW group. The meta-analysis by Hannestad et al. (2011) also found a reduction in IL-1β correlated with better outcomes with SSRIs. Interestingly there was no change in cytokines levels following either exercise dosage. The authors suggest this may have occurred due to pre-treatment with SSRIs – a well known anti-inflammatory agent (Hannestad et al., 2011) – which obscured the ability to detect changes in cytokine levels. Indeed, many past studies have shown exercise to have a robust anti-inflammatory effect in both human and rodent studies (Rethorst et al., 2011; Eyre and Baune, 2012a).

Another recent study by Irwin and Olmstead (2012) utilized a 9-week TCC program in a healthy older adult population to investigate the effect of exercise on depression symptoms. This study found TCC reduced depressive symptoms (BDI) in correlation with a reduction in IL-6 levels. TCC, however, had no effect on cellular markers of inflammation (i.e., sIL-1ra, sIL-6, sICAM, and IL-18). The authors suggest PA treatments may modulate IL-6 via decreasing sympathetic outflow. Aging and stress are associated with increases in circulating catecholamine levels, which are known to increase IL-6.

A study by Kohut et al. (2006) found aerobic exercise reduced pro-inflammatory factors (i.e., CRP, IL-5, TNF-α, and IL-18) more than a combination of flexibility and strength exercise over a 10-month period. These exercise types both reduced depressive symptoms in the Geriatric Depression Scale (GDS).

The robust lipolytic effects of PA are suggested to play a role in the antidepressant effects of PA in depression, via reducing the systemic pro-inflammatory state seen in obesity (Gleeson et al., 2011). A high visceral fat mass has been shown to cause a chronic inflammatory state, and this chronic inflammatory state may link depression and obesity (Stuart and Baune, 2012). Gleeson et al. (2011) also suggests physical inactivity is a risk factor for the accumulation of visceral fat which may predispose individuals to chronic illness like depression and heart disease via systemic PIC production by visceral fat mass.

See Tables 1 and 2 for clinical studies examining the effects of exercise on neuroimmunological factors with and without depressive symptom correlations, respectively.

**Table 1:** **Neuroimmune effects of physical activity in human populations with depressive symptom correlation**.

Study	Study objective	Study details	Exercise details	Neuropsychological testing	Immune testing	Results
Rethorst et al. (2012)	To examine the extent to which inflammatory markers can be used to predict response to exercise treatment after an incomplete response to an SSRI	Prospective. Randomized. TREAD study	Randomized to either 16 or 4 KWW	Clinician: IDS-C30	ELISA of serum at baseline and 12 weeks. IFN-γ, IL-1β, IL-6, and TNF-α	High baseline TNF-α (>5.493 pg/ml) α greater ↓ in depression sxs (IDS-C) over 12 weeks (*p* < 0.0001)
	To examine how the inflammatory markers change with exercise and if those changes are associated with dose of exercise or changes in symptom severity	Participants had MDD and were partial responders to an SSRI (i.e., ≥14 HRSD-17 following >6 weeks but <6 months of treatment)	Aerobic EXC (treadmill or cycle ergometers)	Self-rated: IDS-SR30 and HRSD-17		Sig pos α between Δ IL-1β and Δ depression sxs (*p* = 0.04). For 16KKW not 4 KKW NS change in cytokine levels following 12 weeks of EXC. NS relationship between EXC dose and change in cytokine levels High TNF-α may predict better outcomes with EXC vs. ADs ↓ IL-1β α positive depression treatment outcomes	
		Excluded if regularly engaging in PA Age 18–70 years 73 participants 12-week	Combination of supervised and home-based sessions		
Rethorst et al. (2011)	To determine whether the relationship between IL-6 and depressive symptoms is moderated by participation in moderate-intensity physical activity in a sample of primary care patients	Cross-sectional 97 participants. Family medicine clinic ≥40 years CES-D > 15	Moderate-intensity PA. Measured using modified Community Health Activities Model program for Seniors Activity Questionnaire for older adults	CES-D	ELISA of serum IL-6	Correlation between IL-6 and depressive sxs NS (*r* = 0.086, *p* = 0.40) Association between IL-6 and depressive symptoms was moderated by PA (*p* = 0.02) Among those who did not engage in mod PA, higher depressive sxs α ↑ IL-6 (*r* = 0.28, *p* = 0.05) Association was NS for moderate PA (*r* = −0.13, *p* = 0.38)
Irwin and Olmstead (2012)	To evaluate the effects of a behavioral intervention, TCC on circulating markers of inflammation in older adults	83 healthy older adults (59–86 years) RCT. Two arms – TCC, HE 16 weeks intervention + 9 weeks follow-up	TCC and HE Groups of 7–10 TCC 20 min, 3/week	BDI PSQI	ELISA of plasma for IL-6, CRP, sIL-1ra, sIL-6, sICAM, IL-18 ^NB^*High* *IL-6 > 2.46 pg/ml*	High IL-6 at entry: TCC ↓ IL-6 comparable to those in TCC and HE who had low IL-6 at entry IL-6 in HE remained higher than TCC and HE with low entry IL-6 TCC ns Δ cellular markers of inflammation TCC = ↓ depressive sxs α ↓ IL-6
Kohut et al. (2006)	To determine if a long-term exercise intervention among older adults would reduce serum inflammatory cytokines, and if this reduction would be mediated, in part, by improvements in psychosocial factors and/or by β-adrenergic receptor mechanisms	Adults ≥ 64 years. Community-based Randomized to aerobic or flexibility/strength EXC. 10 months A sub-group of patients on non-selective β1β2-adrenergic antagonists were included	Aerobic (CARDIO) or flexibility/strength EXC (FLEX) 3 days/week, 45 min/day, 10 months	GDS, PSS, CS, SPS, and LOT	ELISA of plasma: CRP, IL-6, TNF-α, and IL-18	EXC = ↓ depressive symptoms, ↑ optimism CARDIO EXC = ↓ IL-6, IL-18, CRP, TNF-α vs. FLEX FLEX EXC = ↓ TNF-α, no change in IL-6, IL-18, CRP ↓ CRP α ↓ depressive symptoms No effect for non-selective β1β2-adrenergic antagonists

*TREAD, treatment with exercise augmentation for depression; KKW, kilocalories per kilogram of body weight per weeks; HE, health education; PSQI, Pittsburgh Sleep Quality Index; GDS, Geriatric Depression Scale; PSS, Perceived Stress Scale; CS, Coherence Scale; SPS, Social Previsions Scale; LOT, Life Orientation Test; α, association with or correlation with; EXC, exercise; IDS-C30, Inventory of Depressive Symptomatology; IL, interleukin; TNF, tumor necrosis factor; IFN, interferon; ELISA, enzyme-linked immunosorbent assay; CRP, C-reactive protein; CES-D. Center for Epidemiologic Studies Depression Scale; NS, non-significant; TCC, Tai Chi Chih*.

#### Neuroimmunological effects in pre-clinical populations

As seen in Tables 3 and 4, there are a large number of studies investigating the neuroimmunological effects of PA. Studies have been variously conducted with and without behavioral correlates. The following section will summarize the salient studies in this field.

A recent study found a voluntary exercise regimen to be associated with increased HC MIF, as well as *Bdnf* and *Tph2* (tryphophan hydroxylase, involved in the synthesis of 5-HT) gene expression (Moon et al., 2012). These changes occurred in the context of reduced depression-like behavior (FST), and the effect of PA on these factors was mediated by the CD74-GTPase (MIF receptor) and RhoA-ERK1/2 pathway. MIF is a PIC expressed in the CNS whose deletion is associated with increased anxiety- and depression-like behaviors, as well as of impaired HC-dependent memory and HC neurogenesis (Conboy et al., 2011). Taken together, this information suggests a role of MIF in mediating the antidepressant action of exercise, probably by enhancing 5-HT neurotransmission and neurogenesis.

Other studies found investigating the effects of PA on neuroimmune-related factors suggest PA increases anti-inflammatory or immunomodulatory factors, e.g., IL-10, IGF-1, and CX3CL1. Sigwalt et al. (2011) shows that in a rat model of depression induced by repeated dexamethasone administration, swimming exercise reduces depression-like behavior in correlation with increased HC IL-10, BDNF, and DNA oxidation. Duman et al. (2009) and Kohman et al. (2012) show voluntary wheel running associated with increased IGF-1, a factor recently shown to have anti-inflammatory effects.

Physical activity has been found to have beneficial effects on immunocompetent glial cells. A study by Latimer et al. (2011) has shown PA to revise age-related astrocyte hypertrophic/reactivity and myelin dysregulation – changes associated with neuroinflammation, cognitive decline, and reduced vascular function. Kohman et al. (2012) recently published a study showing PA attenuates aging associated increases in the proportion of new microglia within the HC (Iba-1 labeled). Furthermore, they show PA increases the pro-neurogenic phenotype of microglia (i.e., IGF-1-releasing microglia) which may contribute to increased HC neurogenesis. Given the robust anti-inflammatory effect of PA, the authors suggest PA may reduce PIC protein production leading to impaired microglial proliferation. A recent study by Barrientos et al. (2011) shows access to a running wheel reduced PIC expression from cultured microglia of aged rats. A recent study by Vukovic et al. (2012) suggests PA enhances the immunomodulatory factor CX3CL1 in the HC, with this associated with enhanced microglia-dependent neural precursor activity, as per the *ex vivo* neurosphere assay.

**Table 2:** **Neuroimmune effects of physical activity in human populations without depressive symptom correlation**.

Study	Study objective	Study details	Exercise details	Immune testing	Results
Nicklas et al. (2008)	To determine the effects of a long-term exercise intervention on two prominent biomarkers of Inflammation, CRP and IL-6, in elderly men and women	Single-blind, randomized, controlled trial	Moderate-intensity PA. Combined aerobic, strength, balance, and flexibility exercise	ELISA of plasma: CRP and IL-6	PA = ↓ IL-6 vs. SA. No ΔCRP
		424 elderly (70–89 years), non-disabled, and community-dwelling men and women	Approx 1 h sessions, 3/week. Starting in center and transition to home-based exercise	
		12 months of moderate-intensity PA vs. successful aging (SA) health education intervention		
Donges et al. (2010)	To determine the effects of 10 weeks of resistance or aerobic exercise training on IL-6 and CRP. Further, to determine pre-training and post-training associations between alterations of IL-6 and CRP and alterations of total body fat mass (TB-FM), intra-abdominal fat mass (IA-FM), and total body lean mass (TB-LM)	102 sedentary subjects Resistance group (RG), aerobic group (AG), or control. 10 weeks	Supervised exercise Control group maintained sedentary lifestyle and dietary patterns	IL-6, CRP	RG and AG = ↓ CRP, no effect on IL-6
		Subjects were involved in DEXA, muscle strength, aerobic fitness measures, and lipid profiling		
Martins et al. (2010)	Effect of exercise on metabolic profile in a healthy elderly sample	RCT *N* = 63 16 weeks	Aerobic: 40–80% HR max Resistance: 8 exercises – 1set/8reps to 3sets/15reps	Total cholesterol, triglycerides – colorimetric end-point assay HDL, LDL – two-point kinetic assay Hs-CRP – immunoturbidometry [@ baseline, 16 weeks]	Aerobic and resistance exercise = improvement in all measures
Stewart et al. (2007)	The purpose of this study was to examine the influence of a 12-week exercise training program on inflammatory cytokine and CRP concentrations. A secondary purpose was to determine whether training-induced changes in cytokines and CRP were influenced by age	29 younger (18–35 years) and 31 old (65–85 years) subjects	Inactive groups complete 12 weeks (3 days/week) of aerobic and resistance exc	ELISA of serum: CRP	Prescribed EXC = ↓ CRP, no change for IL-6, IL-1β, TNF-α for both young and older subjects
		Assigned to young physically active, young physically inactive, older physically active, older physically inactive groups	Physically active control groups continue their normal exc programs	ELISA of plasma: IL-6, TNF-α, and IL-1β	
Black et al. (2012)	To examine if a yogic meditation might alter the activity of inflammatory and antiviral transcription control pathways that shape immune cell gene expression	45 family dementia caregivers Randomized to either Kirtan Kriya Meditation (KKM) or Relaxing Music (RM)	8 weeks of KKM or RM. Both 12-min/day	Genome-wide transcriptional profiles collected from PBMC at baseline and 8 weeks follow-up. RNA extraction ⋄ cRNA Transcript Origin Analysis	KKM = ↑ 19 gene’s expression (immunoglobulin-related transcripts) KKM = ↓ 49 gene’s expression (PIC, activation-related immediate-early genes). From plasmacytoid dendritic cells and B lymphocytes Effects may be due to ↓ NF-κB and IRF-1
Santos et al. (2012)	To assess the effects of moderate exercise training on sleep in elderly people as well as their cytokine profiles	22 male, sed, health, elderly	Mod training for 24 weeks. 60 min/day, 3 days/week	ELISA plasma: TNF-α, IL-6, IL-1, and IL-10	EXC = ↑ aerobic fitness, ↓ REM latency, ↓ time awake
		Polysomnography collected week – 1 and 6	Work rate equiv to ventilator aerobic threshold (VO_2max_, VATI)		EXC = ↓ IL-6, TNF-α, TNF-α/IL-10
		Total body mass and% fat. Whole-body plethysmography			EXC = ↑ IL-10
Cordova et al. (2011)	To investigate the association between long-term RT and circulating levels of the pro-inflammatory mediators IL-6, TNF-α, and IFN-γ in elderly women	Cross-sectional	In RT group women underwent 8.6 ± 0.3 months of EXC.	ELISA plasma: TNF-α, IL-6, and IFN-γ	RT = ↓ IFN-γ, ↓ IL-6, ↓ TNF-α vs. sed
		54 years. Women RT – *N* = 28 Sed – *N* = 26	Mod-intensity (70% 1RM) 50 min, 3/week, 3 sets of 12 reps per exercise		RT = ↓ caloric intake, sBP FFM 1/α IL-6
Libardi et al. (2012)	The aim of the present study was to evaluate the effects of 16 weeks of RT, ET, and CT on inflammatory markers, CRP, and functional capacity in sedentary middle-age men	Healthy inactive subjects. ∼ 49.5 years ± 5	3 weekly sessions for 60 min for 16 weeks	ELISA plasma: TNF-α, IL-6, and CRP	RT and CT = ↑ max strength
		Randomized to RT (*N* = 11), ET (*N* = 12), CT (*N* = 11), or ctrl (*N* = 13)	Max strength (1RM) tested in bench press and leg press		ET and CT = ↑ VO_2peak_
		BMI, waist-to-hip ration, DEXA for FFM	VO_2peak_ measured in incremental exc test		Ns Δ TNF-α, IL-6, CRP
		Diet contents recorded			
Beavers et al. (2010b)	Effect of chronic exercise on inflammation in the elderly	RCT *N* = 424	12 months combined aerobics, strength, flexibility/balance training	CRP, IL-6, IL-6sR, IL-8, and IL-15, Adiponectin, Il-1rα, IL-2sRα, TNF-α, and sTNFRI and II ELISA	Exercise = ↓ IL-8, no Δ in others
Colbert et al. (2004)	Effect of exercise on inflammation in the elderly	Cross-sectional *N* = 3075	Questionnaire	CRP, IL-6, and TNF-α (blood/serum) – ELISA	↑ Exercise α ↓ CRP (*p* < 0.01), ↓ IL-6 (*p* < 0.001), ↓ TNF-α (*p* = 0.02)
Geffken et al. (2001)	Effect of physical activity on inflammation in healthy elderly	Cross-sectional *N* = 5201	Questionnaire	Blood: CRP, fibrinogen, Factor VIII activity, and WCC	↑ Physical activity α ↓ Inflammatory markers
Nybo et al. (2002)	Is prolonged exercise associated with an altered cerebral IL-6 response?	Quasi-experimental *N* = 8, young men Injected with radiotracer (133-Xe)	2 min × 60 min bouts of cycle ergometer at 50% VO_2max_ at different temperatures	Blood: IL-6 – ELISA	Prolonged exercise = ↑ IL-6 release
Kohut et al. (2006)	Effect of different exercise types on inflammation in the elderly	RCT *N* = 87 M34/F53	10 months: 45 min 3×/week	Blood: CRP, IL-6, TNF-α, and IL-18	Cardio = ↓ all markers (*p* < 0.05)
		Subset administered non-selective β-adrenergic antagonists	Cardio: 65–80% VO_2max_		Strength/flex = ↓ TNF-α (*p* = 0.001)
			Strength/flexibility: 10–15 reps (moderate-intensity)		β-inhibitors made no effect
Reuben et al. (2003)	Effect of physical activity on inflammation in elderly	Cross-sectional *N* = 877	Sef-reported: Yale Physical activity survey	Blood: IL-6, CRP – ELISA	↑ Physical activity α ↓ IL-6 and CRP

*RT, resistance training; ET, endurance training; CT, concurrent training; FFM, free fat mass; VATI, ventilator anaerobic threshold; TCC, Tai Chi Chih; RCT, randomized controlled trial; IL, interleukin; TNF, tumor necrosis factor; IFN, interferon; ELISA, enzyme-linked immunosorbent assay; CRP, C-reactive protein*.

**Table 3:** **Neuroimmunological effects of physical activity in rodent populations: with behavioral correlates**.

Study	Study objective	Animal	Exercise type	Behavioral assessment	Immune measures	Results: behavioral	Results: neuroimmune
Moon et al. (2012)	To determine the underlying mechanism of MIF in HC neurogenesis and its role in exercise-induced antidepressant therapy	Rat MIF−/− and WT	Voluntary EXC vs. ECT	FST	*In vivo*: HC, RT-PCR, IB, IHC	*MIF*−*/*− = depression-like behavior	EXC = ↑ *Tph2 in vitro* and *in vivo* (*in vitro* α ↑ 5-HT)
		*In vivo* component	28 days of EXC or 10 days of ECT		*In vitro*: PCR, RT-PCR	*MIF*−*/*− = blunted antidepressant effect of EXC in FST	EXC = ↑ *Bdnf in vitro* and *in vivo*
			ICV injection with MIF *In vitro*: neuronal cell lines treated with MIF. Neuro 2A			Administration of MIF protein = antidepressant effect in FST	CD 74-GPTase (MIF receptor) and RhoA-ERK1/2 pathway mediated MIF-induced Tph2 and Bdnf gene expression and 5-HT content
			MIF −/− = ↓ *Dcx* and *Pax6* siRNAs, GTPase RhoA inhibitor CT04, MEK inhibitor U0126				EXC = ↑ MIF (HC) (IHC and IB)
Sigwalt et al. (2011)	The aim of the present study was to investigate the influence of swimming exercise training on behavior and neurochemical parameters in a rat model of depression induced by repeated dexamethasone administration	Adult Wistar rats. 60 days Daily s.c. dex (1.5 mg/kg) or saline administration	4 groups: CTRL, EXC, DEX, and DEX + EXC	SPT	RIA blood corticosterone	DEX: ↓ sucrose consumption, ↑ immob time	DEX: ↑ HC DNA oxidation, ↑ IL-10, ↑ BDNF, ↓ blood corticosterone levels, ↓ adrenal weight, ↓ body mass
			EXC: swimming/aerobic. 1 h/day, 5 days/week for 3 weeks. Overload of 5% of rat body weight	FST	IHC HC: BDNF 8OHdG	EXC: ↑ sucrose consumption	EXC: normalization of BDNF and IL-10, ↑ blood testosterone, ↓ HC DNA oxidation
			CTRL: fluoxetine 10 mg/kg		RT-PCR HC: BDNF, IL-10	
Duman et al. (2009)	To assess the role of peripheral IGF-I in mediating antidepressant-like behavior under resting physiological conditions	Mice. C57Bl/6	Voluntary wheel running for 4 weeks	FST	PFC and HC	IGF-1 = ↓ immob time, ↑ sucrose consumption	Anti-IGF-1 blocked the BDNF producing effect of EXC
	To investigate the extent to which IGF-I might contribute to antidepressant-like behavior in exercising mice	uCMS		NIH	ELISA for IGF-1	Anti-IGF-1 blocked the antidepressant effect of EXC (FST)	EXC = ↑ IGF-1 mRNA
		IGF-1 and anti-IGF-1 was administered s.c.		SCT	ISH for IGF-1 and BDNF		EXC ≠ PFC IGF-1 mRNA, nor HC and PFC BDNF

*IHC, immunohistochemistry; IB, immunoblot; HC, hippocampus; PFC, pre-frontal cortex; SPT, sucrose preference test; dex, dexamethasone; FST, forced-swim test; MIF, macrophage migration inhibitory factor; RT-PCR, reverse transcription polymerase chain reaction; IB, immunoblot; ELISA, enzyme-linked immunosorbent assay; CTRL, control; BDNF, brain-derived neurotrophic factor; ISH, *in situ* hybridization*.

**Table 4:** **Neuroimmune effects of physical activity in rodent populations: without behavioral correlates**.

Study	Study objective	Animal	Exercise Type	Neuroimmune measures	Results: immune
Funk et al. (2011)	To examine the impact of voluntary exercise on a model of TNF receptor activation dependent neuronal apoptosis	Mice. Pathogen-free CD-1	Voluntary running wheel access for 2 weeks	Flow cytometry of CD11b, CD4, and GFP	EXC = ↓ neuronal death, TNF-α, TNFr1, *MyD88*, TGF-β, CCL2, CCL3
		WT and IL-6−/−		IHC HC GFP+, Iba-1 cells; IL-6, IL-6 Rα, gp130, pAkt, p-STAT3	EXC = ↑ IL-1α mRNA, IL-1RA mRNA, IL-6 (mRNA and protein), neuronal IL-6-Rα
		IP injection of TMT (2.4 mg/kg) or saline		Mass spect: Tin (sn)	TMT = ↑ IL-1α mRNA, IL-1RA mRNA, IL-6 (mRNA and protein), neuronal IL-6-Rα
		Bone-marrow chimera mice used to confirm lack of infiltrating monocytes with TMT injury		Fluorescent microscopy HC for cell death and microglia phenotyping	EXC = ↓ TNF-α cell death signaling pathways with TMT. IL-6 pathway recruitment occurred in both EXC and TMT conditions – IL-6 downstream signal events differed in the level of STAT3 activation
				qPCR	EXC ≠ BDNF mRNA, NGF mRNA, GDNF mRNA
				Microarray analysis: cell death and IL-6 pathways	IL-6−/− mice: EXC showed ↓ neuroprotection against TMT-induced injury
Kohman et al. (2012)	To evaluate whether exercise modulates division and/or activation state of microglia in the dentate gyrus of the hippocampus	Adult (3.5 months) and aged (18 months) BALB/c mice	Vol running wheel for 8 weeks	IHC: BrdU HC	Aged mice = ↑ new microglia
				IF (confocal microscopy): HC: microglia (Iba-1 +), microglial division (Iba-1+ and BrdU +), co-expression of IGF-1, new neuron survival (BrdU × fraction displaying NeuN)	EXC = ↓ new microglia in aged mice, ↑ microglial IGF-1 expression, ↑ survival of new neurons + proliferation EXC ≠ microglial survival or proliferation in adult mice ^NB^*IGF-1-releasing microglia considered pro-neurogenic*
Yi et al. (2012)	To determine if regular treadmill running may blunt the effect of western diet on hypothalamic inflammation	Ldlr−/− (low-density lipoprotein receptor deficiency) and WT mice	Moderate, regular treadmill running exercise. Involuntary. 30 min/day, 5 days/week, 26 weeks	IP glucose tolerance test performed	EXC = ↓ hypothalamic inflammation, ↓ microglial activation
		High-fat diet exposure Indirect calorimetry performed	Exhaustion tests at weeks 0 and 25	Blood glucose levels measured Plasma insulin via ELISA Blood markers: TNF-α, IL-6, INF-g, IL-1α, PAI-1, and MCP-1 IHC: hypothalamus for iba-1	EXC = ↑ glucose tolerance EXC ≠ circulating cytokines
Ehninger et al. (2011)	Effect of exercise on cell genesis in the adult amygdala	Female C57BL6/J mice, 2 mo	Exercise vs. 2 sedentary controls (environmental enrichment, standard housing) 10 days, voluntary wheel running	Iba-1, S100β, BrdU, NeuN, NG2, CNPase, GFAP, and ki67 (hippocampus) – immunofluorescence	Exercise and environmental enrichment = ↑ oligodendroglial precursor proliferation, ↓ microgliogenesis, ↑ neuroplasticity
Latimer et al. (2011)	To test the hypothesis that exercise initiated at mid-age can slow the development of hippocampal glial and vascular biomarkers of early aging	C57BL/6 mice: young, middle and aged	Voluntary exercise for 6 weeks	BP monitoring	EXC = ↓ HC GFAP and MBP which were associated with aging
				IHC HC: astrocyte (GFAP) and myelin staining (MBP)	EXC = astrocytic changes, i.e., fewer branches, finer processes, less hypertrophied
				ELISA HC: VEGF (angiogenesis marker)	EXC = ↑ VEGF which was associated with aging
				Vascular casting: scanning electron micrographs of MCA were utilized	EXC = improved endothelial functioning (less ragged and irregular, ↑ ECN) and ↓ BP
Jeon et al. (2012)	To examine the effects of aging vs. exercise on serum profiles of cytokines and chemokines in mice models	C57BL/6 mice. Young (2 months) and old (20 months)	Forced treadmill exc for 4 weeks. 30 min/day, 5 days/week	Multiplexed bead-based sandwich immunoassay of 50 serum cytokines/chemokines	Treadmill EXC ≠ Δ serum cytokines/chemokines significantly
					Older mice = ↑ eotaxin, IL-9, TARC vs. young mice
Wu et al. (2012)	Effect of exercise on hippocampal neurogenesis in infection	Male/female IL-1β^XAT^ (IL-1β over-expression) C57BL/6 mice, 8–12 months vs. WT	Exercise vs. sedentary control	MHCII DCX, BrdU, Iba-1 (HC) – immunohistochemistry	EXC ≠ normalized neurogenesis in presence of centrally mediated infection in IL-1β over-expression
			Intra-hippocampal FIV (feline immunodeficiency virus) injection vs. vehicle 2 weeks, voluntary wheel running	
Nichol et al. (2008)	Effect of exercise on amyloid load and neuroinflammation in AD mice	Male/female Tg2576 C57B16/SJL mice, 16–18 months vs. WT	Exercise vs. sedentary control 3 weeks, voluntary wheel running	HC and cortex	EXC = ↓ TNF-α, IL-1β
				Pro-inflammatory: IL-1β, TNF-α – ELISA	EXC = ↑ IFN-γ, CD11c, MHCII, CD40, MIP-1α
				Adaptive/alternate immune markers: IFN-γ, CD40, MHCII – Western blot	EXC = ↑ CD68, mannose receptor
				CD11c, MIP-1α – Immunohistochemistry	(↑ Perivascular MΦ infiltrate)
				Aβ – ELISA, Dot-blot analysis	
				CD68, mannose receptor – Immunohistochemistry	
				Iba-1 – Western blot	
Vukovic et al. (2012)	Effect of exercise on microglial-dependent hippocampal neurogenesis	Female TG-Csf1r-GFP C57BL/6J mice, 6–8-week old	Exercise vs. sedentary control *Ex vivo* neurospehere culture with/without microglia 2 weeks, voluntary wheel running	HC BrdU, DCX, Iba-1 – immunostaining CX3CL1 – ELISA MHCII – FACS	EXC = microglial-dependent ↑ neural precursor activity EXC = ↓ MHCII+ve microglia, EXC = ↑ CX3CL1 (neuroprotective phenotype)
Ziv et al. (2006)	Role of immune cells in neurogenesis	Male Sprague Dawley rats, 12-week old	EE vs. standard lab control	From HC: IHC: BrdU, MHCII, IB-4, IGF-1, NeuN, BDNF, and TCR	EE = ↑ neurogenesis and adaptive microglial profile in presence of function T-cell population
			Healthy rats vs. immune-deficient (SCID mice)	
Leem et al. (2011)	Effect of exercise in neuroinflammation in AD mice	Male/Female Tg-Ad (NSE/htau23) C57BL/6 mice 16 months vs. WT	EXC vs. sedentary control	From HC	High intensity EXC = ↓ phsophoTau (*p* < 0.05)
			Intermediate (12 m/min) vs. high intensity exercise (19 m/min)	RT-PCR: TNF-α, IL-6, and IL-1β	High intensity EXC = ↓ gliosis [MAC-1, GFAP] (*p* < 005)
				WB: iNOS, ERK, COX-2, p38	High intensity EXC = ↓ μAPK-dependent signaling pathway [↓ iNOS, TNF-α, IL-6, IL-1β] (*p* < 0.05)
				IHC: phosphoTau, GFAP, MAC-1, and p65	
Herring et al. (2012)	Effect of exercise in pregnancy on AD pathology in offspring	Female Tg-AD APP695 CRND8 x C57BL/6-C3H-HeJ vs. WT	Exercise vs. sedentary control Duration of pregnancy, voluntary wheel running	From entire brain, except, cerebellum, brainstem	EXC = ↓ Aβ in offspring via altered APP processing (*p* < 0.022)
				IHC: Aβ, A1F1, laminin, RELN	EXC = ↑ angiogenesis (*p* < 0.022)
				RT-PCR: Gapdh, APP, Lpap1, ApoE1, Clu, A2m, Mmp9, Mme	EXC = ↑ neuroplasticity
				DC protein assay: Aβ_40_, Aβ_42_, sAPPα	EXC = ↓ microgliosis (*p* = 0.002), pro-inflammatory mediators, oxidative stress mediators (*p* = 0.029)
				WB: APP, CTFβ, RELn, APOER2, VLDR, ADC, CYP, IDE, IBA-1, PTGER2, SOD1, SOD2	
Carmichael et al. (2010)	Role of brain MΦ on central cytokines and fatigue post-exercise	Male C57Bl/6 mice, 8-week old	Exercise vs. sedentary control	IL-1β (cerebrum) – ELISA	EXC = ↑ IL-1β from MΦs
			MΦ depletion with clodronate injection or saline Single bout of exercise, 22 m/min for 150 m	

*ECN, endothelial cell nuclei; EE, environmental enrichment; IHC, immunohistochemistry; WB, western blot; TCR, T-cell receptor; IL, interleukin; TNF, tumor necrosis factor; IFN, interferon; ELISA, enzyme-linked immunosorbent assay; CRP, C-reactive protein; EXC, exercise; APP, amyloid precursor protein; TMT, trimethyltin; NGF, nerve growth factor, BDNF, brain-derived neurotrophic factor; VEGF, vascular endothelial growth factor*.

A study by Funk et al. (2011) demonstrates that PA can offer significant protection to the HC in a chemical-induced injury model [via trimethyltin (TMT)] that involves TNF receptor signaling. PA attenuated TMT-induced changes such as loss of DG neurons and microglial activation. Furthermore, PA was accompanied by a significant elevation in IL-6 and IL-1ra mRNA levels and repressed elevations in PICs and chemokines (CCL2 and CCL3). Interestingly, the investigators identified a functional role for IL-6 in neuroprotection given mice deficient in IL-6 (IL-6 knock-out) were not responsive to the neuroprotective effects of PA on the HC. The effects of PA and TMT on IL-6 downstream signal events differed at the level of STAT3 activation. The beneficial effects of acute spikes in IL-6 with PA is clearly a significant factor in the anti-inflammatory effect of PA. In a human study by Starkie et al. (2003), 3 h of cycling blunted the endotoxin-induced increase in circulating TNF-α levels, and this effect was mimicked by an IL-6 infusion. Further, this regulatory role of IL-6 on TNF-α levels was demonstrated in anti-IL-6 treated mice and IL-6 knock-out mice (Mizuhara et al., 1994; Matthys et al., 1995). Whilst acute elevations in IL-6 are found throughout the body (Funk et al., 2011), a recent study shows a selective increase in IL-6 localized to the HC (Rasmussen et al., 2011).

Neuroimmune cells may also have a role in the beneficial effects of PA. A study by Ziv et al. (2006) found PA, a component of the EE protocol, was associated with enhanced HC neurogenesis alongside a neuroprotective microglia phenotype and in the presence of a T-cell population. The role of CNS-specific T cells in the neuroprotective effects of PA is suggested given severe combined immunodeficiency (SCID) mice exposed to EE did not show an increase in neurogenesis.

## Model of Neuroimmunological Effects of PA in Depression

Emerging evidence suggests the neuroimmune system is critical in both the development of depression-related pathophysiology and in the treatment of depression. From the evidence available in this field, PA has a multitude of beneficial neuroimmune effects which may lead to the improvement of depression-related neurobiological processes, hence leading to reduced depression-like behaviors.

**Figure 2:**
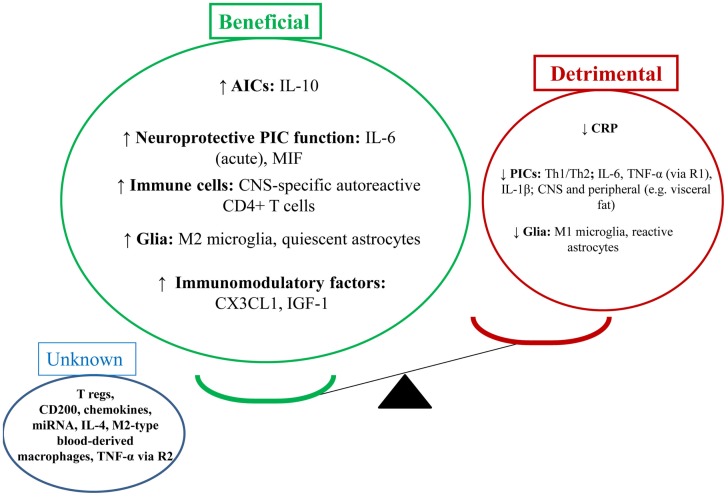
**Physical activity in depression: antidepressant via enhancing the beneficial effects of the neuroimmune system**. This figure illustrates the effects of PA on the brain as per the balance between beneficial and detrimental effects of neuroimmune factors. PA appears to enhance the beneficial effects of the neuroimmune system and reduce the detrimental effects. From a behavioral perspective, this may lead to reduced depression-like behaviors. From a clinical perspective, this may lead to reduced depressive symptoms, depressive episode resolution, and reduced relapse rates (disease prevention).

From a neuroimmune perspective, evidence suggests PA does enhance the beneficial and reduce the detrimental effects of the neuroimmune system. Figure 2 outlines these effects. PA appears to increase the following factors: IL-10, IL-6 (acutely), MIF, CNS-specific autoreactive CD4+ T cells, M2 microglia, quiescent astrocytes, CX3CL1, and IGF-1. On the other hand, PA appears to reduce detrimental neuroimmune factors such as: Th1/Th2 balance, PICs, CRP, M1 microglia, and reactive astrocytes. The effect of other factors is unknown, such as: T regs, CD200, chemokines, miRNA, M2-type blood-derived macrophages, and TNF-α (via R2). The beneficial effects of PA are likely to occur centrally and peripherally (e.g., in visceral fat reduction).

Based on the strong relationship between the neuroimmune system and other neurobiological systems (i.e., neuroplasticity, neuroendocrine function, and neurotransmission), we believe PA may exert beneficial behavioral effects via these neurobiological systems. PA’s neuroimmune effects are likely involved in enhanced neuroplasticity, reduced oxidative stress, increases in 5-HT, dopamine, and noradrenaline, and enhanced glucocorticoid sensitivity.

The neurobiological effects of PA – mediated largely via the neuroimmune system – are likely involved with reduced depression-like behaviors in rodents (i.e., sickness-like behavior, anhedonia, anxiety-, and cognition-like behaviors) and positive clinical effects (i.e., reduced depressive symptoms, enhanced cognitive function, relapse reduction, and early intervention).

## Discussion

Physical activity is increasingly investigated as a preventative, early intervention, and treatment option in depression. The interest in investigation of PA may have arisen for a number of reasons: the burden of depression is rising so novel therapeutic and preventative options are required (WHO, 2008; Berk and Jacka, 2012; Cuijpers et al., 2012; Southwick and Charney, 2012). Rates of physical inactivity are high and rising in modern society (Lee et al., 2012) with early evidence suggesting a link to the development of depression (Pasco et al., 2011a,b). Pharmacotherapy in depression is hampered by relatively high rates of resistance (Rush et al., 2006a,b) and considerable side-effects. Evidence is emerging to suggest co-morbid links between obesity, diabetes, heart disease, and depression (Baune and Thome, 2011; Stuart and Baune, 2012), and PA is a therapeutic option with beneficial cardio-metabolic effects (Gleeson et al., 2011; Baune et al., 2012c; Hamer et al., 2012; Knochel et al., 2012; Stuart and Baune, 2012).

Based on the abovementioned factors, research has been reviewed to better understand the clinical efficacy of different types of PA, to understand the mechanism of action of PA and to investigate for suitable biomarkers to measure the treatment effect of PA in depression. Further, a model has been suggested in order to assist in understanding the neuroimmune effects of PA in depression.

An important consideration in the field of exercise immunology includes understanding the mechanisms of treatment response in depression vs. other psychiatric disorders. At present the authors feel there is no enough data to address this issue systematically, with research evidence. Whilst it would appear that the effects of PA on the immune system in various disorders – in both clinical and pre-clinical studies – is quite similar, i.e., PICs are reduced (particularly in anxiety disorders and depression; Gleeson et al., 2011; Eyre and Baune, 2012a), this considers only a narrow range of neuroimmune factors. The authors speculate that the therapeutic difference in PA may occur due to subtle variations in the neuroimmune and neurobiological effect, dependent upon the CNS environment with each pathophysiological state. Studies investigating the effects of a standardized exposure to PA, in various psychiatric disorders in parallel, may assist in unraveling this complex issue.

When considering the balance between the beneficial and detrimental effects of immune system and the effect of PA tipping this balance toward beneficial effects, it is important to consider: *Is it possible to restore the balance of the immune system and still suffer from a low mood?* This is an interesting question and open to debate. It would seem that the majority of evidence suggests that as inflammation increases, mood worsens, and as inflammation reduces, mood appears to return to normal. For example, this is shown in meta-analysis by Dowlati et al. (2010) and a review by Maes (2011) whereby depressive symptoms are associated with elevations in PIC levels. Another meta-analysis shows inflammation reduces with the use of SSRIs in the treatment of depression (Hannestad et al., 2011). However, there are other therapies such as SNRIs which appear to improve mood, yet have no effect on levels of inflammation (Hannestad et al., 2011). Therefore, more work is required to understand the effect of various therapies (pharmacological and non-pharmacological) on a wider variety of immune-related factors such as cytokines (anti- and pro-inflammatory), anti-inflammatory factors like IGF-1, CD200, CX3CL1, MIF, neuroprotective systemic immune cells, etc. Interestingly, Walker (2012) suggests the concentration of antidepressant drug molecules in the CNS also alters the immunomodulatory effects.

### Future directions

From current evidence, it is not possible to ascertain the type of PA which is most efficacious in the treatment of depression. Although, most evidence surrounds aerobic exercise. We suggest the need for head-to-head clinical trials comparing different types and intensities of PA to assist in making this issue clearer. Moreover, when considering the effects of distinct types of PA on neuroimmune factors, we also suggest the need for more head-to-head clinical trials (Baune and Eyre, 2012).

The most recent study examining the effects of PA on depressive symptoms was conducted by Rethorst et al. This study suggests that a high baseline TNF-α level was associated with a greater reduction in depressive symptomatology as opposed a high baseline TNF-α level being a negative factor for SSRI efficacy (Hannestad et al., 2011; Rethorst et al., 2012). The authors suggest TNF-α levels may be a moderator between SSRI and exercise treatment, and may have a role in personalized treatment algorithms. Whilst this is a promising suggestion, further research is needed to replicate these findings.

Our understanding of the neuroimmune effects of PA in depression will continue to develop as the understanding of the neuroimmune effects of PA develop. It is important to consider the use of multi-biomarker methods within this area in order to better understand potential biomarkers. For example, the use of neuroimaging, serum protein and genetic markers, and behavioral analysis. This type of methodology is increasingly employed in biological psychiatry (Baune et al., 2010, 2012a,b).

There are a number of neuroimmune-related factors which are yet to be considered in the effect of PA in depression. These factors include micro ribonucleic acid (miRNA), neuroimmune-related Positron Emission Tomography (PET) ligands, the neuroprotective effects of neuroimmune factors, and immune cells. Evidence is emerging to suggest a role for miRNAs, factors involved in regulating gene expression at the post-translational level, in modulating the effects of the immune system (Ponomarev et al., 2012). For example, various miRNAs such as miR-155 and miR-124 may have a role in polarizing microglia toward pro- or anti-inflammatory phenotypes, respectively (Ponomarev et al., 2012). The PET ligand, Translocator Protein (TPSO) ligand [(11)C]PBR28, a marker of microglial activation, was recently found to be elevated by LPS-induced systemic inflammation in non-human primates (Hannestad et al., 2012). This ligand has the potential to be utilized as a biomarker to investigate if activation of microglia may be a mechanism through which systemic inflammatory processes influence the disease course of depression. The biology of centrally migrating immune cells and CNS immune cells in depression is complex and far from understood. Regarding the debated issue of blood-derived macrophages can enter the brain parenchyma: research and development into novel methods for permanent differential labeling of circulating monocytes, as contrasted with resident microglia, is underway (Prinz et al., 2011). Studies are required to better understand the role of protective immunosurveillance in clinical and rodent models of depression.

## Conclusion

The investigation of the neuroimmune effects of PA on depression and depression-like behavior is a rapidly developing and important field. This paper summarizes the most recent findings in the area and proposes a model whereby PA enhances the beneficial effects of the neuroimmune system and reduces the detrimental effects of the neuroimmune system.

## Conflict of Interest Statement

The authors declare that the research was conducted in the absence of any commercial or financial relationships that could be construed as a potential conflict of interest.
